# Whole-genome sequencing of Russian poplars to understand relationships within the genus *Populus* L.

**DOI:** 10.3389/fpls.2025.1706329

**Published:** 2025-12-18

**Authors:** Elena V. Borkhert, Elena N. Pushkova, George S. Krasnov, Yuri A. Nasimovich, Ramil A. Murataev, Peter M. Evlakov, Andrey V. Klimov, Marina V. Kostina, Boris V. Proshkin, Daiana A. Krupskaya, Alexey A. Dmitriev, Nataliya V. Melnikova

**Affiliations:** 1 Engelhardt Institute of Molecular Biology, Russian Academy of Sciences, Moscow, Russia; 2 Faculty of Biology, Lomonosov Moscow State University, Moscow, Russia; 3 Voronezh State University of Forestry and Technologies named after G.F. Morozov, Voronezh, Russia; 4 InEcA-Consulting, Novokuznetsk, Russia; 5 West Siberian Branch of the V.N. Sukachev Institute of Forestry SB RAS – branch of the Federal Research Center “Krasnoyarsk Scientific Center SB RAS”, Novosibirsk, Russia; 6 Institute of Biology and Chemistry, Moscow Pedagogical State University, Moscow, Russia; 7 Kuzbass Institute of the Federal Penal Service of Russia, Novokuznetsk, Russia; 8 Moscow Institute of Physics and Technology, Moscow, Russia

**Keywords:** *Populus*, Russian poplars, hybrids, whole-genome sequencing, phylogenetic analysis

## 1 Introduction

Trees of the genus *Populus* L. are important both economically and ecologically. They are used to produce high-quality plywood, paper, pulp, bioplastics, and biofuels, as well as for urban landscaping and phytoremediation (Devappa et al., 2015; Porth and El-Kassaby, 2015; Kobolkuti et al., 2019; Melnikova et al., 2019; Huang et al., 2022; Kang and Wei, 2022). Poplars are also promising subjects for the genetic research of trees due to their small genome sizes (about 400 Mb for *Populus trichocarpa* (Gao et al., 2025)) and ability to reproduce vegetatively and grow rapidly (Taylor, 2002). Numerous studies on poplars have identified genes that affect wood quality and could be modified to enhance these trees’ economically significant traits (Wierzbicki et al., 2019; Buell et al., 2023; Boerjan and Strauss, 2024; Zhu and Li, 2024). Using genotypes carrying valuable allelic variants of genes in breeding is also promising (Biselli et al., 2022). Meanwhile, the frequent hybridization of related *Populus* species, as well as the high level of intraspecific polymorphism, complicates taxon identification and classification (Roe et al., 2014; Jiang et al., 2016). Poplars form several syngameons, which are groups of closely related species with a common gene pool (Cronk and Suarez-Gonzalez, 2018; Nasimovich et al., 2019). This makes *Populus* species an interesting model for studying evolution. Research on the genetic diversity of *Populus* has contributed to our understanding of their phylogenetic relationships and the advisability of distinguishing several sections (Melnikova et al., 2021; Liu et al., 2022; Wang et al., 2022). Genetically, *Populus* species outside of Russia have been relatively well-studied, with numerous whole-genome sequencing results published (several thousand *Populus* samples were sequenced, and the data were deposited in the NCBI SRA database, www.ncbi.nlm.nih.gov/sra/). However, there is practically no whole-genome sequencing data available for Russian poplars (Melnikova et al., 2021). Only the results of targeted deep sequencing for a representative sample set have been presented by us (Borkhert et al., 2023). To obtain a complete picture of the genetic diversity of the genus *Populus*, it is necessary to study Russian poplars and compare them with poplars from other countries. Whole-genome sequencing is the most suitable method for this purpose.

## 2 Materials and methods

### 2.1 Plant material

From 2019 to 2024, we compiled a collection of plant material from 23 genotypes belonging to 11 taxa of the genus *Populus*. Leaf samples were collected from the Russian species *P. laurifolia*, *P. nigra*, *P. suaveolens*, and *P. longifolia*, the latter of which is an adventitious species of unknown origin. Additionally, we collected material from the most common hybrids used in urban landscaping: *P*. × *petrovskoe*, *P*. × *rasumovskoe*, and *P*. × *sibirica*. These hybrids account for about 78% of all poplars in Moscow (Murataev, 2024). We also collected material from other taxa growing in Russia. A list of the collected plant material organized by sections is provided below and in the **Supplementary Table S1**. The detailed description of the studied taxa can be found in the supplementary material in our previous study (Borkhert et al., 2023).

Section *Tacamahaca* Spach:

*P. balsamifera* L. – two samples of a male clone from the Dmitrovka village in the Taldomsky District of the Moscow Region. Initially identified as *P. trichocarpa*, the identification was changed based on the results of the genetic analysis performed in the present study;

*P. laurifolia* Ledeb. – two samples from the natural range (Novokuznetsk);

*P. longifolia* Fisch. (now *P. × longifolia*) – two samples from Moscow representing male and female genotypes of this adventive species;

*P. suaveolens* Fisch. – three samples from the natural range: two from Irkutsk and one from Novokuznetsk;

*P.* × *wobstii* R.I. Schrod. [*P. laurifolia* × *P. longifolia*] – one sample from Moscow.

Section *Aigeiros* Duby:

*P*. *deltoides* W. Bartram ex Marshall – two samples: one from Moscow and one from Sevastopol;

*P.* × *canadensis* Moench – three samples: two from Moscow and one from Voronezh (labeled in the plant nursery as *P.* × *canadensis* var. *regenerata* Rehder);

*P. nigra* L. var. *nigra* – four samples: two from Novokuznetsk, one from Voronezh (from a plant nursery where it was brought from the Hopersky Reserve), and one from Bashkortostan.

Intersectional hybrids *Aigeiros* × *Tacamahaca*:

*P.* × *petrovskoe* R.I. Schrod. ex Wolkenst. – two samples from Moscow;

*P.* × *rasumovskoe* R.I. Schrod. ex Wolkenst. – one sample from Moscow;

*P.* × *sibirica* G.V. Krylov et G.V. Grig. ex A.K. Skvortsov – one sample from Moscow.

### 2.2 DNA extraction and sequencing

The Magen HiPure Plant DNA Mini Kit (Magen, Guangzhou, China) was used for DNA extraction. We assessed the quality and concentration of the DNA using the agarose gel electrophoresis and Qubit 4.0 fluorometer (Thermo Fisher Scientific, Waltham, MA, USA).

A Tn5-based protocol was used to prepare the DNA for whole-genome sequencing (Picelli et al., 2014; Pushkova et al., 2023). The DNA libraries were evaluated using the capillary electrophoresis on Qsep1-Plus (BiOptic, New Taipei City, Taiwan) and fluorometry on Qubit 4.0 (Thermo Fisher Scientific). Sequencing was performed on NovaSeq X Plus (Illumina, San Diego, CA, USA) in a 150 + 150 bp format and HiSeq 2500 (Illumina) in a 125 + 125 bp format.

## 3 Preliminary data analysis

We sequenced the genomes of 23 *Populus* samples from Russia, which represent 11 different taxa. From 2.8 to 12.9 Gb (from 9.5 to 51.6 mln reads) per sample were obtained. The statistics and results of the quality assessment of the obtained sequencing data are presented in **Supplementary Table S2**. For the first time, whole-genome sequencing data were obtained for three Russian taxa: *P. longifolia*, *P.* × *rasumovskoe*, and *P.* × *wobstii*. Three other taxa have been studied outside of Russia (see **Supplementary Table S3**); however, samples from Russia were not previously whole-genome sequenced. These taxa included two samples of *P. laurifolia* from Novokuznetsk, three samples of *P.* × *canadensis* from various locations within the country, and four samples of *P. nigra* from different regions of Russia. *P. suaveolens* has been studied previously, but only one Russian sample has been examined (see **Supplementary Table S3**). We sequenced three samples of *P. suaveolens* from its natural range in Siberia. Additionally, we sequenced samples of *P.* × *petrovskoe* and *P.* × *sibirica*, which represent the cultivated flora of Moscow (Mayorov et al., 2020), and two other species found in Russia: *P*. *deltoides* and *P. balsamifera*.

We conducted a joint study of the whole-genome sequencing data of Russian poplars obtained by us and NCBI SRA *Populus* samples from other countries that we chose as the most representative. Thus, we analyzed 23 our own samples and 74 samples from NCBI SRA (**Supplementary Table S3**), representing the main systematic groups of *Populus*. We paid particular attention to American poplars (*P*. *balsamifera*, *P*. *deltoides*, and *P. trichocarpa*) because they could be the parental species of Russian cultivars. This enabled us to test the hypothesis that natural poplar species are grouped by not only systematic units (subgenera, sections, and subsections), but also by singameons, which are groups of geographically close species with a common gene pool (Nasimovich et al., 2019; Borkhert et al., 2023). Identification of the parental species of *P. longifolia*, which was previously considered an adventive Russian species of unknown origin, was of particular interest.

Illumina reads obtained in the present study and those downloaded from NCBI SRA were first processed using Trimmomatic 0.39 (Bolger et al., 2014) with the following parameters: TRAILING:28, SLIDINGWINDOW:4:17, and MINLEN:40. Also, residual adapters were removed with Trimmomatic 0.39 (ILLUMINACLIP:adapters.fa:2:30:10:8:TRUE). We mapped the processed reads to the *P. trichocarpa* “Stettler 14” genome v1.1 (https://phytozome-next.jgi.doe.gov/info/PtrichocarpaStettler14_v1_1) (Hofmeister et al., 2020) using BWA-MEM 0.7.17 (Li and Durbin, 2009) with slightly increased sensitivity (-k 17 parameter). Secondary alignments were then removed using samtools (view -F 2048) (Danecek et al., 2021), and reads with any soft-clipped bases were completely removed using awk. On average, 73% (from 69% to 83%) of reads were mapped, except for one sample (*P. × canadensis* Voronezh_548), for which only 24% of reads were mapped (**Supplementary Table S2**). For this sample, fungal and bacterial contamination was detected (https://trace.ncbi.nlm.nih.gov/Traces/?view=run_browser&acc=SRR35248035&display=analysis). However, since we only used reads that mapped to the *P. trichocarpa* genome in further analysis, this should not affect the results. For the remaining samples (obtained from NCBI SRA), the mapping rate was 64% on average and varied from 21% to 88%, depending on the analyzed species.

Subsequently, duplicate reads were marked using FixMateInformation and MarkDuplicatesWithMateCigar (Picard-tools 2.21.3, https://broadinstitute.github.io/picard/). The proportion of duplicate reads ranged from 61% to 65% (including an average of 7% optical duplicates), except for the one sample mentioned above. Variant calling was then performed using Freebayes 1.3.1 (minimum mapping Q 25, minimum base Q 20, minimum alternate allele coverage 4, minimum total coverage 5, minimum variant allele frequency 0.2) (Garrison and Marth, 2012). The search was restricted to gene coding regions (CDS). Variants with Phred Q greater than 25 were included in the subsequent analysis (lists of DNA polymorphisms were deposited to Zenodo, https://zenodo.org/records/17123067).

Then, we calculated pairwise Euclidean distances between per-sample variant allele frequency (VAF) vectors (**Supplementary Table S4**). For this procedure, only positions covered by at least 8 reads in both samples in a pair were considered; otherwise, the position was excluded for this pair. The distances (more precisely, the expression under the square root) were normalized to the number of positions included in the analysis for a pair. Next, we performed clustering using Ward’s method (ward.D2) and visualized the results. Bootstrapping (1000 replications) was done using the ‘shipunov’ 1.17.1 package (R 4.2.2) (https://www.r-project.org/). PPLine (Krasnov et al., 2015) was used to perform all the described above analyses.

**Figure 1:**
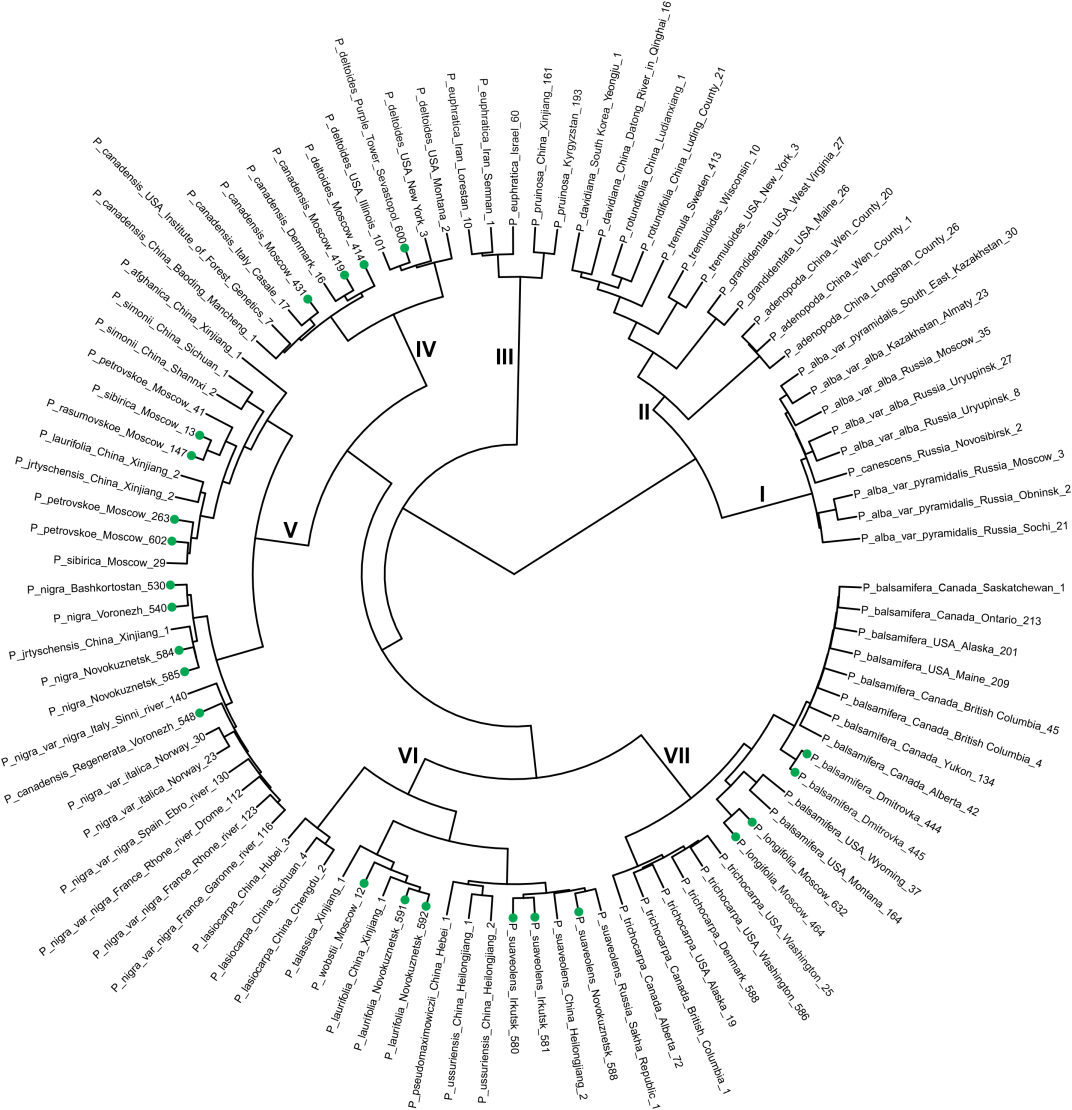
Dendrogram of 97 *Populus* samples based on the whole-genome sequencing data. DNA polymorphisms (VAF values) in gene sequences (CDS) were analyzed. The genotypes sequenced by us are marked by green dots.

**Figure 1** shows the dendrogram obtained for the analyzed *Populus* samples (see also **Supplementary Figure S1**, which represents the same dendrogram in a linear view and with bootstrap analysis). Genotypes sequenced by us are marked by green dots. As can be seen, our samples and previously studied samples were arranged in a highly logical manner, i.e., strictly according to subgenera, sections, and subsections. This indicates that the analysis accurately reflects the systematics of taxa within the genus *Populus*. The only exception was *P. simonii*, which was placed near *P. nigra* and its hybrids (Cluster V). However, based on its morphological characteristics, we have previously noted the intersectional nature of *P. simonii* (Nasimovich et al., 2019), which could be implicated in this result.

Additionally, though less clearly, species were distributed by singameons. For example, aspen species from Eurasia, including *P. tremula*, *P. davidiana*, and *P. rotundifolia*, were found to be closely related in cluster II. North American aspen species, such as *P. tremuloides* and *P. grandidentata*, were distinct from Eurasian aspens, while *P. adenopoda* from southern China was distinct from all of them (cluster II). This species differs from the others in this subfamily in terms of its morphological characteristics as well (Tsarev, 1985).

The singameon of representatives of section *Tacamahaca* from North America (*P. balsamifera* and *P. trichocarpa*) was clearly separated in cluster VII, and the samples were divided into species groups. Two samples from Russia that were initially identified by us as *P. trichocarpa* (Dmitrovka_444 and Dmitrovka_445) were found to belong to the main group of *P. balsamifera*. However, both these trees were male and lacked capsules. Without capsules, *P. trichocarpa* and *P. balsamifera* are nearly indistinguishable (Rehder, 1949). Thus, the analysis allowed us to refine the systematic classification of these samples.

The singameon of Eurasian poplars was particularly well-separated. These were representatives of section *Tacamahaca* (*P. laurifolia*, *P. suaveolens*, and others) and section *Leuce* Duby (*P. lasiocarpa*) in cluster VI. The Eastern species of “balsam” poplars, *P. suaveolens*, as well as the closely related species *P. ussuriensis* and *P. pseudomaximowiczii*, which were recently classified as *P. suaveolens* (Eckenwalder, 1996; Skvortsov and Belyanina, 2006), formed a distinct subcluster among the “balsam” poplars of Eurasia in cluster VI. The Western poplar species (*P. laurifolia* and others) formed their own subcluster in cluster VI. Thus, geographical location, along with systematic affiliation, is one of the factors that determine the taxon’s genetic characteristics.

*P.* × *petrovskoe*, *P.* × *rasumovskoe*, and *P.* × *sibirica* – intersectional hybrids of *P. nigra* and Eurasian “balsam” poplars – formed a separate group in cluster V. This group clustered with *P. nigra*. Thus, the studied hybrids are genetically related to Russian poplars, which was not obvious until recently. For example, *P.* × *sibirica* was believed to be a cross between North American *P. balsamifera* and Russian *P. nigra* (Skvortsov, 2007, Skvortsov, 2010). In our previous study (Borkhert et al., 2023), we identified the ancestor species for *P.* × *petrovskoe*, *P.* × *rasumovskoe*, and *P.* × *sibirica*, but there were no American poplars in that analysis. The present results reasserted our previous findings.

*P. longifolia*, a Russian species of unknown origin, clustered with *P. balsamifera* (Cluster VII), rather than with *P. trichocarpa* or *P. suaveolens*. However, until recently, it was believed that *P. longifolia* was closely related to the latter two species (Skvortsov, 2008; Mayorov et al., 2012). At the same time, *P. longifolia* showed great similarity to *P. suaveolens* and its hybrids according to the genetic distances (**Supplementary Table S4**). These results support the hypothesis that *P. longifolia* is a hybrid from the crossing of *P. balsamifera* and *P. suaveolens*. A similar hypothesis was proposed by us earlier (Kostina and Nasimovich, 2014). This hypothesis is also supported by the morphological evidence such as the presence of a mixture of naked 2- and 3-valved capsules in *P. longifolia*, while *P. balsamifera* has naked two-valved capsules and *P. suaveolens* has naked three-valved capsules (Vasilieva et al., 2018). The hybridization of *P. balsamifera* and *P. suaveolens* could have likely occurred in a botanical garden, given that these species grow on different continents.

Thus, our study filled a gap in the whole-genome sequencing data on poplars grown in Russia. These data can be used to establish relationships between *Populus* species and hybrids, study hybrid formation, and evaluate the intraspecific diversity of poplars. Additionally, the data allow one to assess the DNA polymorphism in specific genes, including those associated with economically valuable traits and essential processes. This is a promising approach for selection of best poplar genotypes for specific applications based on DNA markers. The obtained sequencing data also form the basis for developing DNA markers to identify poplar species and hybrids. Identifying them based on morphological characteristics requires a highly qualified specialist and can only be done when the poplars have leaves and, in some cases, fruits, so this is an urgent issue.

## Data Availability

The data presented in the study are deposited in the NCBI SRA repository, accession number PRJNA1314865.

## Supplementary material

Supplementary Figure 1 Dendrogram of 97 *Populus* samples based on the whole-genome sequencing data.

Supplementary Table 1 List of 23 *Populus* samples sequenced in the present study.

Supplementary Table 2 Statistics of the whole-genome sequencing data obtained for the 23 *Populus* samples.

Supplementary Table 3 List of 74 *Populus* samples for which WGS data were downloaded from the NCBI SRA (www.ncbi.nlm.nih.gov/sra/).

Supplementary Table 4 Genetic distances between the 97 studied *Populus* samples based on the whole-genome sequencing data.
