# Supplementary material for: Whole-genome sequencing of Russian poplars to understand relationships within the genus Populus L
Source: Front Plant Sci. 2025 Dec 18;16:1706329. doi: 10.3389/fpls.2025.1706329 (PMC12756557; doi:10.3389/fpls.2025.1706329)

Supplementary Figure S1. Dendrogram of 97 *Populus* samples based on the whole-genome sequencing data. DNA polymorphisms (VAF values) in gene sequences (CDS) were analyzed. 1000 bootstrap replications were used.

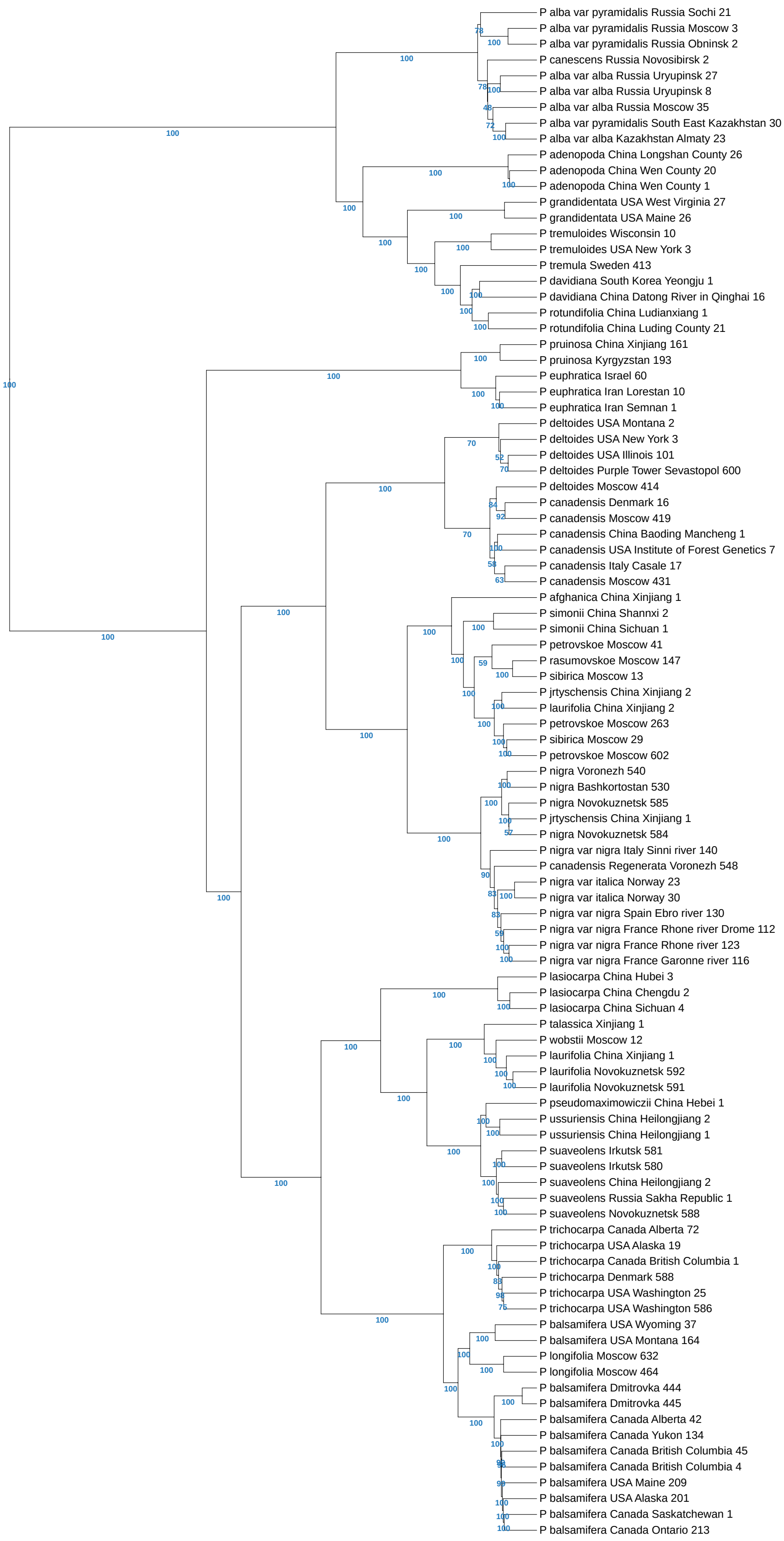

Supplement: Supplementary Figure 1 — Dendrogram of 97 Populus samples based on the whole-genome sequencing data. [file Image1.pdf]
