# Supplementary material for: Whole-genome sequencing of Russian poplars to understand relationships within the genus Populus L
Source: Front Plant Sci. 2025 Dec 18;16:1706329. doi: 10.3389/fpls.2025.1706329 (PMC12756557; doi:10.3389/fpls.2025.1706329)
Supplement: Supplementary Table 1 — List of 23 Populus samples sequenced in the present study. [file Table1.docx]

**Supplementary Table S1.** List of 23 *Populus* samples sequenced in the present study.

Twenty-three samples belonging to 11 taxa of the genus *Populus* L. were studied.

**Sect. *Tacamahaca* Spach**

*P. balsamifera*, Dmitrovka, 444. Initially identified as *P. trichocarpa*, but the species was clarified based on the results of genetic analysis. Russia, Moscow Region, Taldom District, Dmitrovka Village 15.

*P. balsamifera*, Dmitrovka, 445. Initially identified as *P. trichocarpa*, but the species was clarified based on the results of genetic analysis. Russia, Moscow Region, Taldom District, Dmitrovka Village 15. Coordinates: 56.749222 N, 37.738840 E.

*P. laurifolia*, Novokuznetsk, 591. Russia, Kemerovo Region, Novokuznetsk.

*P. laurifolia*, Novokuznetsk, 592. Russia, Kemerovo Region, Novokuznetsk.

*P. longifolia*, Moscow, 464. Russia, Moscow. Coordinates: 55.75644 N, 37.43122 E.

*P. longifolia*, Moscow, 632. Russia, Moscow, Shchukinsky peninsula.

*P. suaveolens*, Irkutsk, 580. Russia, Irkutsk Region, Slyudyanka. Coordinates: 51.644431 N, 103.704007 E.

*P. suaveolens*, Irkutsk, 581. Russia, Irkutsk Region, Vydrino village. Coordinates: 51.644431 N, 103.704007 E.

*P. suaveolens*, Novokuznetsk, 588. Russia, a tree grown in Novokuznetsk from a cutting brought from Chukotka.

*P. × wobstii* (Moscow poplar, which is close to *P. × wobstii* and *P. laurifolia*), Moscow, 12. Russia, Moscow.

**Sect. *Aigeiros* Duby**

*P. deltoides*, Moscow, 414. Russia, Moscow. Coordinates: 55.834755 N, 37.538887 E.

*P. deltoides*, ‘Purple Tower’, 600. Russia, Sevastopol, Orlinoye village.

*P. × canadensis*, Moscow, 419. Russia, Moscow. Coordinates: 55.820208 N, 37.563317 E.

*P. × canadensis*, Moscow, 431. Russia, Moscow. Coordinates: 55.811016 N, 37.572927 E.

*P. × canadensis* ‘Regenerata’, Voronezh, 548. Russia, Voronezh, VGLTU plant nursery.

*P. nigra*, Bashkortostan, 530. Russia, Republic of Bashkortostan. Coordinates: 56.134038 N, 54.150389 E.

*P. nigra*, Voronezh, 540. Russia, cuttings brought from the Hopersky Reserve and planted at VGLTU in Voronezh.

*P. nigra*, Novokuznetsk, 584. Russia, Kemerovo Region, Novokuznetsk.

*P. nigra*, Novokuznetsk, 585. Russia, Kemerovo Region, Novokuznetsk.

**Sect. *Aigeiros* × Sect. *Tacamahaca***

*P. × petrovskoe*, Moscow, 263. Russia, Moscow.

*P. × petrovskoe*, Moscow, 602. Russia, Moscow.

*P. × rasumovskoe*, Moscow, 147. Russia, Moscow.

*P.*× *sibirica*, Moscow, 13. Russia, Moscow.
