# Supplementary material for: Whole-genome sequencing of Russian poplars to understand relationships within the genus Populus L
Source: Front Plant Sci. 2025 Dec 18;16:1706329. doi: 10.3389/fpls.2025.1706329 (PMC12756557; doi:10.3389/fpls.2025.1706329)
Supplement: Supplementary Table 3 — List of 74 Populus samples for which WGS data were downloaded from the NCBI SRA (www.ncbi.nlm.nih.gov/sra/). [file Table3.docx]

**Supplementary Table S3.** List of 74 *Populus* samples for which WGS data were downloaded from the NCBI SRA (www.ncbi.nlm.nih.gov/sra/).

**Sect. *Populus***

**Subsect. *Populus* (*Albidae* Dode)**

***P. alba* L. subsp. *alba*.** Eurasia (from Central and Southern Europe to Western Siberia and Central Asia).

*P. alba* var. *alba*, 8. Russia: Uryupinsk, Khopyor river valley. SRX24508057

*P. alba* var. *alba*, 27. Russia: Uryupinsk, Khopyor river valley. SRX24508038

*P. alba* var. *alba*, 23. Kazakhstan: South-East Kazakhstan, Almaty. SRX24508042

*P. alba* var. *alba*, 35. Russia: Moscow. SRX24508029

***P. alba* L. subsp. *pyramidalis* (Bunge) W. Wettst.** (*P. bolleana* Lauche).

*P. alba* var. *pyramidalis*, 2. Russia: Obninsk.SRX24508063

*P. alba* var. *pyramidalis*, 3. Russia: Moscow. SRX24508062

*P. alba* var. *pyramidalis*, 21. Russia: Sochi. SRX24508044

*P. alba* var. *pyramidalis*, 30. Kazakhstan: South-East Kazakhstan. SRX24508035

**Subsect. *Trepidae* Dode**

***P. davidiana* Dode (*P. tremula* var. *davidiana* (Dode) Hulten. (Primorye, etc.).**

*P. davidiana*, 1. South Korea: Yeongju. SRX19841534

*P. davidiana*, 16. China: Datong River in Qinghai. SRX8189657

***P. sieboldii* Miq. (*P. rotundifolia* Simon-Louis, not Griff.; *P. tremula* var. *sieboldii* (Miq.) Kudo).** Eurasia (practically only Japan, but also the southernmost part of Sakhalin). Otherwise – *P. tremula* var. *sieboldii* (Miq.) H. Onoshi.

*P. rotundifolia*, 1. Ludianxiang, Yulong Naxi Autonomous County, Lijiang, Yunnan. SRX10573663

*P. rotundifolia*, 21. China: Luding County, Ganzi Tibetan Autonomous Prefecture, Sichuan. SRX7913313

***P. tremula* L.** Eurasia (Europe, Western Asia, Siberia) and North Africa.

*P. tremula*, 413. Sweden: Simlang. SRX1359657

***P. tremuloides* Michs.** North America (from Labrador to Alaska, south to New Mexico and California).

*P. tremuloides*, 3. USA: New York. SRX9751761

*P. tremuloides*, 10. USA: Wisconsin, Waushara country. SRX1362154

***P. grandidentata* Michx.** North America (from southern Saskatchewan to Ontario and Minnesota, south to North Carolina, Tennessee, Illinois, and Iowa).

*P. grandidentata*, 26. USA: Maine. SRX9751750

*P. grandidentata*, 27. USA: West Virginia. SRX9751739

***P. adenopoda* Maxim.** Eurasia (Central and Western China).

*P. adenopoda*, 1. China: Wen County, Gansu, Longnan, Gansu. SRX10573645

*P. adenopoda*, 20. China: Wen County, Gansu, Longnan, Gansu. SRX10573624

*P. adenopoda*, 26. China: Longshan County, Xiangxi Tujia and Miao Autonomous Prefecture, Hunan. SRX7913315

**Subsect. *Populus* × Subsect. *Trepidae***

***P.* × *canescens* (Aiton) Sm. (*P. alba* × *P. tremula*).** Eurasia (Europe, Western Asia).

*P. × canescens*, 2. Russia: Novosibirsk. Central Siberian Botanic Garden. SRX24508064

**Sect. *Turanga* Bge**

***P. euphratica* Oliver.** Eurasia (from Western to Central Asia) and North Africa.

*P. euphratica*, 1. Iran: Semnan. SRX2933502

*P. euphratica*, 10. Iran: Lorestan. SRX2933494

*P. euphratica*, 60. Israel. SRX2933444

***P. pruinosa* Schrenk.** Eurasia (Central Asia).

*P. pruinosa*, 161. China: Xinjiang. SRX2762410

*P. pruinosa*, 193. Kyrgyzstan. SRX2762378

**Sect. *Leucoides* Spach**

***P. lasiocarpa* Oliv.** Eurasia (Central and Western China).

*P. lasiocarpa*, 2. China: Chengdu. SRX10036877

*P. lasiocarpa*, 3. China: Hubei. SRX9751769

*P. lasiocarpa*, 4. China: Sichuan. SRX9751763

**Sect. *Tacamahaca* Spach**

***P. balsamifera* L. (*P. tacamahaca* Mill.).** North America (from Labrador to Alaska, south to New York, Michigan, Nebraska, Nevada, and Oregon).

*P. balsamifera*, 1. Canada: Saskatchewan. SRX21104101

*P. balsamifera*, 4. Canada: British Columbia. SRX21104098

*P. balsamifera*, 37. USA: Wyoming. SRX21104042

*P. balsamifera*, 42. Canada: Alberta. SRX21104025

*P. balsamifera*, 45. Canada: British Columbia. SRX21104021

*P. balsamifera*, 134. Canada: Yukon. SRX21103851

*P. balsamifera*, 164. USA: Montana. SRX21103784

*P. balsamifera*, 201. USA: Alaska. SRX21103633

*P. balsamifera*, 209. USA: Maine. SRX9751791

*P. balsamifera*, 213. Canada: Ontario. SRX8766490

***P. laurifolia* Ledeb.** Eurasia (southern Western Siberia, Altai, northern Mongolia).

*P. laurifolia*, 1. China: Xinjiang.SRX9751800

*P. laurifolia*, 2. China: Xinjiang. SRX9751734

***P. simonii* Carriere (*P. przewalskii* Maxim.).** Eurasia (Northern China).

*P. simonii*, 1. China: Sichuan. SRX9751807

*P. simonii*, 2. China: Shanxi. SRX9751806

***P. talassica***

*P. talassica*, 1. China: Xinjiang. SRX9751742

***P. trichocarpa* Torr. et Gray ex Hock.** North America (from Alaska and British Columbia to southern California).

*P. trichocarpa*, 1. Canada: British Columbia. SRX21104087

*P. trichocarpa*, 19. USA: Alaska. SRX21104049

*P. trichocarpa*, 25. USA: Washington. SRX21104037

*P. trichocarpa*, 72. Canada: Alberta. SRX21103945

*P. trichocarpa*, 586. USA: Washington.2020. SRX9751792

*P. trichocarpa*, 588. Denmark. SRX9009430

***P. suaveolens* Fisch.** Eurasia (in the narrow sense – Eastern Siberia).

*P. suaveolens*, 1. Russia: Republic of Sakha. SRX9751782

*P. suaveolens*, 2. China: Heilongjiang. SRX9751747

***P. pseudomaximowiczii*.**

*P. pseudomaximowiczii*, 1. China: Hebei. SRX9751729

***P. ussuriensis* Komarov.** Eurasia (Primorye).

*P. ussuriensis*, 1. China: Heilongjiang. SRX9751758

*P. ussuriensis*, 2. China: Heilongjiang. SRX9751730

**Sect. *Aigeiros* Duby**

***P. afghanica*.** Eurasia.

*P. afghanica*, 1. China: Xinjiang. SRX9751764

***P.*× *canadensis* Moench (*P. deltoides* × *P. nigra*).**

*P.*× *canadensis*, 1. China: Baoding, Mancheng. SRX21032719

*P.*× *canadensis*, 7. USA: Davis, California. SRX10653104

*P.*× *canadensis*, 16. Denmark. SRX9009432

*P.*× *canadensis*, 17. Italy: Casale. SRX3577080

***P. deltoides*** **W. Bartram ex Marshall.** North America.

*P. deltoides*, 2. USA: Montana. SRX9751816

*P. deltoides*, 3. USA: New York. SRX9751805

*P. deltoides*, 101. USA: Illinois. SRX3577078

***P. nigra* L. (*P. nigra* var. *nigra*).** Eurasia (Europe, Western Asia).

*P. nigra*, 112. France: Rhone river, Drome. SRX1496639

*P. nigra*, 116. France: Garonne River. SRX1496635

*P. nigra*, 123. France: Rhone river. SRX1496628

*P. nigra*, 130. Spain: River Ebro. SRX1496621

*P. nigra*, 140. Italy: Sinni river. SRX1496611

***P. pyramidalis* Rozier (*P. nigra* var. *italica* Du Roi).**

*P. pyramidalis*, 23. Norway. ERX8951733

*P. pyramidalis*, 30. Norway. ERX8951726

**Sect. *Aigeiros*** **×** **Sect. *Tacamahaca***

***P.*× *irtyschensis* Chang Y. Yang.** Natural hybrid of *P. laurifolia* × *P. nigra* var. *nigra.* Eurasia (Western Siberia).

*P.*× *irtyschensis*, 1. China: Xinjiang. SRX9751740

*P.*× *irtyschensis*, 2. China: Xinjiang. SRX9751731

***P.*× *petrovskoe* R.I. Schrod. ex Wolkenst.** A hybrid that originated spontaneously in Moscow and is widely cultivated. The parent species have not been reliably identified, but we believe them to be *P.* × *canadensis* and *P. laurifolia*.

*P.*× *petrovskoe*, 2. Russia: Moscow. Incorrectly listed in NCBI as *P.*× *sibirica*. SRX9416302

***P.*× *sibirica* G. V. Krylov et G. V. Grig. ex A. K. Skvortsov.** A cultivar widely distributed in Russia. Its parent species have not been reliably identified, but we believe them to be *P. nigra*, *P. laurifolia*, and *P. suaveolens*.

*P.*× *sibirica*, 1. Russia: Moscow. SRX9416303
